# Sex Pheromone Mediates Resource Partitioning Between *Drosophila melanogaster* and *D. suzukii*

**DOI:** 10.1111/eva.70042

**Published:** 2024-11-11

**Authors:** Charles A. Kwadha, Guillermo Rehermann, Deni Tasso, Simon Fellous, Marie Bengtsson, Erika A. Wallin, Adam Flöhr, Peter Witzgall, Paul G. Becher

**Affiliations:** ^1^ Department Plant Protection Biology, Chemical Ecology Group Swedish University of Agricultural Sciences Alnarp Sweden; ^2^ CBGP, INRAE, CIRAD Institute Agro, IRD, University Montpellier Montpellier France; ^3^ Department Natural Science, Design and Sustainable Development Mid Sweden University Sundsvall Sweden; ^4^ Department Biosystems and Technology Swedish University of Agricultural Sciences Lomma Sweden

**Keywords:** chemical communication, competition avoidance, food partitioning, fruit fly, insect–yeast interaction, integrated pest management, invasive species, semiochemicals

## Abstract

The spotted‐wing drosophila, *Drosophila suzukii* and the cosmopolitan vinegar fly *D. melanogaster* feed on soft fruit and berries and widely overlap in geographic range. The presence of *D. melanogaster* reduces egg‐laying in *D. suzukii*, possibly because *D. melanogaster* outcompetes *D. suzukii* larvae feeding in the same fruit substrate. Flies use pheromones to communicate for mating, but pheromones also serve a role in reproductive isolation between related species. We asked whether a *D. melanogaster* pheromone also modulates oviposition behaviour in *D. suzukii*. A dual‐choice oviposition assay confirms that *D. suzukii* lays fewer eggs on blueberries exposed to *D. melanogaster* flies and further shows that female flies have a stronger effect than male flies. This was corroborated by treating berries with synthetic pheromones. Avoidance of *D. suzukii* oviposition is mediated by the female *D. melanogaster* pheromone (*Z*)‐4‐undecenal (Z4‐11Al). Significantly fewer eggs were laid on berries treated with synthetic Z4‐11Al. In comparison, the male pheromone (*Z*)‐11‐octadecenyl acetate (cVA) had no effect on *D. suzukii* oviposition. Z4‐11Al is a highly volatile compound that is perceived via olfaction and it is accordingly behaviourally active at a distance from the source. *D. suzukii* is known to engage in mutual niche construction with the yeast *Hanseniaspora uvarum*, which strongly attracts flies. Adding Z4‐11Al to fermenting *H. uvarum* significantly decreased *D. suzukii* flight attraction in a laboratory wind tunnel and a field trapping assay. That a *D. melanogaster* pheromone regulates oviposition in *D. suzukii* demonstrates that heterospecific pheromone communication contributes to reproductive isolation and resource partitioning in cognate species. Stimulo‐deterrent diversion or push‐pull methods, building on combined use of attractant and deterrent compounds, have shown promise for control of *D. suzukii*. A pheromone that specifically reduces *D. suzukii* attraction and oviposition adds to the toolbox for *D. suzukii* integrated management.

## 1 Introduction

Sex pheromones serve, first of all, mate communication, but may also regulate heterospecific interactions. Sex signals that enhance mate finding and diminish, in addition, hybrid matings between cognate species are under combined sexual and natural selection (Blows 2002; Ritchie 2007). A heterospecific role of pheromones has been shown in moths where sex pheromones have been studied comprehensively (Roelofs and Brown 1982; Witzgall et al. 1996; Baker 2008; El‐Sayed 2023; Fouchier et al. 2023), and also in *Drosophila* (Higgie, Chenoweth, and Blows 2000; Higgie and Blows 2007, 2008; Khallaf et al. 2020). A change in the composition of cuticular hydrocarbon blends involved in mate finding, in response to sympatric populations of another species, has been demonstrated in the sibling species *D. birchii* and *D. serrata* . Reinforcement of mate recognition in zones of overlap generates this reproductive character displacement, leading to reproductive isolation (Higgie, Chenoweth, and Blows 2000; Higgie and Blows 2007).

Associated with the human global expansion, *D. melanogaster* is a cosmopolitan commensal (Arguello, Laurent, and Clark 2019; Sprengelmeyer et al. 2020). Aided by the importation of fresh berries, the spotted‐wing drosophila, *D. suzukii* has spread all over within 15 years and has become economically most important, since it oviposits on ripe and unripe berries (Walsh et al. 2011; Cini et al. 2014; Asplen et al. 2015; Kwadha et al. 2021).

Invaders face competition from established species sharing the same resources. Although *D. suzukii* oviposits on fruit and berries during earlier phenological stages than *D. melanogaster* (Atallah et al. 2014; Keesey, Knaden, and Hansson 2015; Ramasamy et al. 2016), larvae co‐occur and interact in food substrates, where *D. melanogaster* outcompetes *D. suzukii* (Dancau et al. 2017; Shaw et al. 2018; Rombaut et al. 2023). This leads to the question of whether *D. suzukii* can avoid contact with *D. melanogaster* .

*D. suzukii* is attracted to (Hamby et al. 2012; Mori et al. 2017; Jones et al. 2022; Kleman et al. 2022) and engages in niche construction with the fruit‐associated yeast *H. uvarum* , which protects larval substrates against fungal infestations (Chakraborty et al. 2022). Low ethanol production, compared with other yeasts, matches the low ethanol tolerance of *D. suzukii* larvae (Stamps et al. 2012; Buser et al. 2014; Chakraborty et al. 2022). Avoidance of microbiota associated with other species is a possible mechanism to evade competition between fruit‐feeding drosophilds (Rombaut et al. 2023). However, *D. suzukii* feeds on many fruits and berries, in a wide range of habitats (Bühlmann and Gossner 2022; Olazcuaga et al. 2022; Guay et al. 2023), which entails a wide variation in the composition of associated microbiota (Jones et al. 2022; Koerte et al. 2020).

Insect‐produced compounds, in comparison, are more specific and reliable mediators of interaction between competing species, since they are independent of the food substrate. And, there is indeed growing evidence that chemical cues from different life stages of *D. melanogaster* deter oviposition in *D. suzukii* (Dancau et al. 2017; Shaw et al. 2018; Snellings et al. 2018; Kidera and Takahashi 2020; Kienzle and Rohlfs 2021; Tungadi et al. 2022, 2023; Rombaut et al. 2023).

If pheromones play a heterospecific role, the ensuing question is whether male or female pheromones of *D. melanogaster* modulate oviposition behaviour in *D. suzukii*. The male *D. melanogaster* pheromone (*Z*)‐11‐octadecenyl acetate (cVA) is not a species‐specific signal, since it is shared by many drosophilid flies (Bartelt, Schaner, and Jackson 1985; Schaner, Bartelt, and Jackson 1987; Schaner et al. 1989; Hedlund et al. 1996; Khallaf et al. 2021). While cVA modulates mating in *D. melanogaster* (Ejima et al. 2007; Kurtovic, Widmer, and Dickson 2007; Lebreton et al. 2014) and inhibits mating in *D. suzukii* (Dekker et al. 2015), there is no indication that it would deter oviposition in female flies.

*D. melanogaster* females, on the other hand, produce a cuticular hydrocarbon (*Z,Z*)‐7,11‐heptacosadiene (7,11‐HD), that is perceived via contact chemoreceptors at close range to enhance courtship in conspecific males and to inhibit courtship in males of the sibling species *D. simulans* (Billeter et al. 2009; Billeter and Wolfner 2018; Kohl, Huoviala, and Jefferis 2015; Auer and Benton 2016). Apart from *D. melanogaster* , 7,11‐HD has only been found in two island‐endemic *Drosophila* and in trace amounts in the cosmopolitan species *D. virilis* , among other dienic cuticular hydrocarbons (Jackson and Bartelt 1986; Khallaf et al. 2021).

It is probably difficult to test 7,11‐HD in isolation, since autoxidation continuously affords aldehydes, including (*Z*)‐4‐undecenal (Z4‐11Al), which is a strong attractant for conspecific *D. melanogaster* males and females, and has an antagonistic effect on the attraction of *D. simulans* (Lebreton et al. 2017). Both 7,11‐HD and Z4‐11Al enhance courtship in *D. melanogaster* males (Billeter et al. 2009; Borrero‐Echeverry et al. 2022), but only Z4‐11Al is active over a distance, since it is highly volatile and perceived via an olfactory receptor, DmelOr69aB. Traces of Z4‐11Al left by females on surrounding substrates, or even on males during mating, become a public message that is perceptible even by the human nose (Lebreton et al. 2017; Frey et al. 2022).

We therefore asked whether the *D. melanogaster* female sex pheromone Z4‐11Al mediates heterospecific interactions with *D. suzukii*. We show that Z4‐11Al has an antagonistic behavioural effect on *D. suzukii*, both at a distance and at close range. Z4‐11Al impaired female flight attraction to the yeast *H. uvarum* , in a wind tunnel and field trapping assay and reduced oviposition on blueberries.

## 2 Materials and Methods

### 2.1 Insects

*D. melanogaster* , Dalby strain (Sweden) (Lebreton et al. 2012) and an Italian strain of *D. suzukii* (Revadi et al. 2015) were maintained on a standard sugar syrup‐yeast‐cornmeal medium at 25°C ± 2°C, RH 50% ± 5%, and a photoperiod of 12:12 (L:D). Emerging flies were collected 3–6 h post‐eclosion. Flies were immobilized with CO_2_ during 2–4 min and sexed under a microscope. Males and females were kept separately: 2–4‐days‐old *D. melanogaster* and 4–6‐days‐old *D. suzukii* flies were used for experiments. *D. suzukii* were mated 2–4 h after onset of the photophase, and single mating pairs were transferred to clean vials. Mated females were kept 1–2 h in the vial before testing (Pitnick, Markow, and Spicer 1995; Revadi et al. 2015).

### 2.2 Chemicals

(*Z*)‐4‐undecenal (Z4‐11Al) was synthesized (Lebreton et al. 2017): chemical and isomeric purity were > 99% and 98.6%, respectively. (*Z*)‐11‐octadecenyl acetate (Z11‐18Ac; cVA) was purchased from PheroBank (Wijk bij Duurstede, Netherlands): (*E*)‐2‐undecenal (E2‐11Al) was a gift from E. A. Wallin (Sundsvall, Sweden): chemical and isomeric purity were > 99%, respectively. Synthetic compounds were diluted in ethanol.

### 2.3 Dual Choice Oviposition Assays

Ripe blueberries ( *Vaccinium corymbosum* L.), were obtained from a local grocery shop. Berries were rinsed with distilled water before use. Only berries with blue‐coloured pulp were used (Little, Chapman, and Hillier 2018).

In the first assay, two berries of similar weight (±0.1 g) were placed in a Petri dish (Ø 115 × 65 mm; VWR). To assess if exposure to *D. melanogaster* induces oviposition avoidance in *D. suzukii*, blueberries were pre‐exposed to three or 10 mated *D. melanogaster* males (*n* = 24 and 21, respectively) or unmated females (*n* = 20 and 23, respectively) in polystyrene Drosophila vials (Ø 25 × 95 mm; Fisher Scientific) during 2 h. Berries kept in vials without *D. melanogaster* were used as a control. A single gravid female *D. suzukii* was then added into each Petri dish and eggs laid into the two blueberries were counted after 24 h. An oviposition index (OI) was calculated, the quotient of the differential and the sum of eggs, laid on treatment and control berries (OI = eggs control—eggs treatment/eggs control + eggs treatment).

Similarly, for establishing whether aversion is induced by *D. melanogaster* pheromone, we tested pairs of berries treated with either 5 ng Z4‐11Al or 5 μL ethanol (*n* = 34), and berries with 5 ng cVA or 5 μL ethanol (*n* = 49). For comparison, headspace collections of *D. melanogaster* females contained ca. 3 ng Z4‐11Al per female (Lebreton et al. 2017). A control assay compared untreated berries with berries treated with 5 μL ethanol. A subsequent dose–response test included berries treated with 5 μL ethanol and either 0.5 ng (*n* = 36), 5 ng (*n* = 34) or 50 ng (*n* = 39) Z4‐11Al. Additional control experiments were done with 5 μL ethanol vs. 5 μL ethanol or untreated berries, respectively, and with 5 ng Z4‐11Al vs. 5 ng of the positional isomer E2‐11Al (*n* = 34).

We used a second dual choice oviposition assay to establish if aversion to Z4‐11Al is olfactory. Five mated *D. suzukii* females were introduced in a BugDorm cage (30 × 30 × 30 cm; Megaview, Taiwan), where one berry was placed on top of each two 30‐mL polypropylene cups (Nolato Cerbo AB, Trollhätan, Sweden). The cup lids were made of a fine fabric net that allowed emission of volatiles from inside the cup while restricting flies from entering the cups. Berries were either exposed to volatiles of Z4‐11Al (100 ng) or the solvent ethanol (100 μL) dispensed from 1.5‐mL microcentrifuge tubes held in upright position inside the cups. After 7 h, flies were removed, and eggs laid on each berry were counted to determine the OI (*n* = 25 cages).

### 2.4 *Hanseniaspora uvarum* Culture

The yeast *Hanseniaspora uvarum* is often found in association with *D. suzukii* (Hamby et al. 2012; Chakraborty et al. 2022). Following Kleman et al. (2022), colonies of *H. uvarum* grown on potato dextrose agar (PDA; Difco) (39 g/L) were used to establish liquid pre‐cultures in potato dextrose broth (PDB; Difco) (24 g/L). An aliquot of 3 mL from 1‐day‐old preculture was inoculated in 50 mL PDB in 100‐mL fermentation flasks (Duran‐Group, Mainz, Germany). Both pre‐cultures and cultures were maintained in a shaking incubator at 25°C and 260 rotations/min for 24 h.

### 2.5 Wind Tunnel Assay

A glass wind tunnel with a 100 × 30 × 30 flight section (Becher et al. 2010) was used to test the effect of Z4‐11Al on upwind flight attraction to *H. uvarum* yeast volatiles. Mated 4–6‐days‐old *D. suzukii* females that had been starved for 6 h were flown individually to fermenting *H. uvarum* headspace, alone or blended with Z4‐11Al (*n* = 73/stimulus). Charcoal‐filtered air (0.4 L/min) was blown through a wash bottle containing 30 mL of a *H. uvarum* culture that had been inoculated 20–24 h before testing. The outlet was a teflon tube (Ø 0.5 cm) placed at the upwind end of the tunnel. A piezo sprayer (El‐Sayed, Gódde, and Arn 1999) delivered ethanol at a rate of 10 μL/min (control), or synthetic Z4‐11Al at 10 ng/min, dissolved in 10 μL/min ethanol, into the yeast plume. The outlet from the wash bottle and the sprayer were placed side‐by‐side to merge plumes in the centre of a glass cylinder (12 cm Ø × 10 cm), aligned with wind direction. The downwind end of the glass cylinder was covered by a metal mesh (pore size 2 × 2 mm): females flying upwind and approaching this metal mesh (< 5 cm) were scored. Air blown by a horizontal fan into the wind tunnel was filtered with active charcoal elements. Glassware and metal mesh were decontaminated in an oven during 8 h at 350°C.

### 2.6 Field Trapping

For field trapping, red Drososan traps (Koppert Biological Systems) were baited with *H. uvarum* in liquid medium (PDB) and synthetic Z4‐11Al was released from an open 1.2‐mL glass vial, held with a wire above the yeast bait, inside the trap (Kleman et al. 2022). The four treatments comprised 30 mL liquid PDB medium and 1 mL ethanol (control); 30 mL PDB and 960 ng Z4‐11 Al in 1 mL ethanol; *H. uvarum* in 30 mL PDB and 1 mL ethanol; *H. uvarum* in 30 mL PDB and 960 ng Z4‐11 Al in 1 mL ethanol. Traps were placed in a quadrangular arrangement with 5 m distance between the traps at three sites in Montevideo, Uruguay (*n* = 5/site). After 2 days, traps were collected and the captured drosophilid flies were identified.

### 2.7 Statistical Analysis

All analyses were calculated with R software (R Core Team 2021), at α = 0.05. The R package ‘lme4’ (Bates et al. 2015) was used for generalized linear mixed models (GLMM). To test normality of residuals from GLMMs, we used a Shapiro–Wilk test. The number of eggs laid was analysed by a GLMM fitted with a binomial distribution. We considered pre‐exposure and treatment with synthetic chemicals as a fixed effect, while fly (replicates) and day were considered random effects. The flight assay was analysed by GLMM fitted with a binomial distribution, with stimulus and day considered as fixed and random effects, respectively. Field captures were analysed by a GLMM fitted with a Poisson distribution followed by Tukey's contrast pairwise comparison between the different treatments (R package ‘multcomp’; Hothorn, Bretz, and Westfall 2008).

## 3 Results

### 3.1 *D. suzukii* Lays Fewer Eggs on Blueberries Exposed to *D. melanogaster* Flies

**FIGURE 1:**
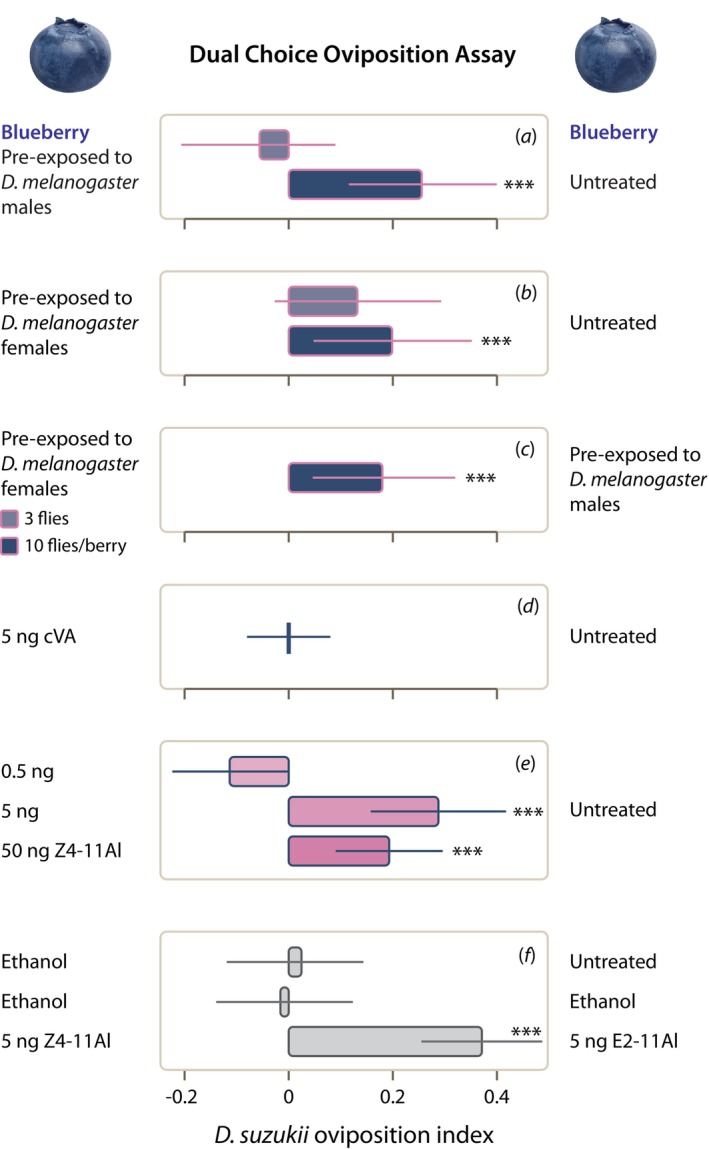
Antagonistic effect of *Drosophila melanogaster* flies and synthetic sex pheromone Z4‐11Al on *D. suzukii* egg‐laying, in a dual choice oviposition assay. The oviposition index is the quotient, of the differential and the sum, of the eggs laid on the control and the treated blueberry. A positive oviposition index shows that more eggs were laid on the control berry (right‐hand side). (a, b) Blueberries were exposed to 3 and 10 *D. melanogaster* male or female flies. Compared with untreated berries, *D. suzukii* females laid significantly fewer eggs on berries pre‐exposed to 10 males or 10 females. (c) In a direct comparison, *D. suzukii* females laid fewer eggs on berries that were pre‐exposed to females rather than males. (d) Synthetic cVA, a *D. melanogaster* male pheromone, had no effect, (e) while 5 ng and 50 ng of the female pheromone Z4‐11Al decreased oviposition. (f) Ethanol had no effect on oviposition preference, and significantly more eggs were laid on berries treated with the unsaturated aldehyde E2‐11Al, compared to Z4‐11Al. Error bars show standard errors: asterisks show significance, according to GLMM fit by maximum likelihood (***p < 0.001).

In a first dual choice oviposition assay, *D. suzukii* females laid fewer eggs on berries that had been exposed to 10 mated *D. melanogaster* males or females, compared with untreated control berries (Figure 1a; *n* = 21, *Z* = 4.65, *p* < 0.001; Figure 1b, *n* = 23, *Z* = 4.9, *p* < 0.001). Exposure to 3 flies did not have a significant effect (Figure 1a,b). A direct comparison between berries exposed to 10 males and 10 females, showed that berry exposure to *D. melanogaster* mated females had a stronger effect on *D. suzukii* oviposition avoidance than exposure to *D. melanogaster* mated males (Figure 1c; *n* = 23, *Z* = −4.75, *p* = 0.002).

### 3.2 Female *D. melanogaster* Pheromone Z4‐11Al Induces Oviposition Avoidance in *D. suzukii*

We next asked whether contamination of berries with *D. melanogaster* male or female pheromones caused a reduction in *D. suzukii* egg‐laying.

*D. melanogaster* males transfer cVA to females during mating, which reduces the response of males to freshly mated females (Billeter and Wolfner 2018). *D. suzukii*, unlike many other drosophilid species, does not produce the male aphrodisiac pheromone cVA, but *D. suzukii* females carry a dedicated olfactory receptor, and they perceive and respond to cVA (Dekker et al. 2015). Likewise, *D. melanogaster* females transfer Z4‐11Al to mating males (Frey et al. 2022) and *D. suzukii* expresses an ortholog of the receptor tuned to Z4‐11Al in *D. melanogaster* (Lebreton et al. 2017; Walker et al. 2023).

Accordingly, the antagonistic effect of *D. melanogaster* mated males and females on *D. suzukii* oviposition (Figure 1a,b) could have been due to either male or female pheromone. Treating berries with synthetic pheromones confirms the result of a direct comparison of berries exposed to *D. melanogaster* males and females (Figure 1c). Synthetic male pheromone cVA did not affect *D. suzukii* egg‐laying at all (Figure 1d), whereas 5 and 50 ng of the female pheromone Z4‐11Al induced significant oviposition avoidance (Figure 1e; *n* = 34, *Z* = −2.38, *p* = 0.001; *n* = 39, *Z* = −4.05, *p* = 0.005).

Berries treated with 5 ng of another unsaturated aldehyde, (*E*)‐2‐undecenal (E2‐11Al) were strongly preferred for oviposition in a choice test with 5 ng of Z4‐11Al. Ethanol, used as solvent for synthetic compounds, had no effect on oviposition (Figure 1f; *n* = 34, *Z* = 3.32, *p* = 0.001).

Finally, another dual choice assay was done to establish whether aversion to Z4‐11Al is olfactory. Berries were placed on netted cups, emanating Z4‐11Al diluted in ethanol (100 ng Z4‐11Al per cup) or ethanol alone. Significantly more eggs were laid on berries exposed to ethanol alone (oviposition index 0.19 ± 0.1; *n* = 25, *Z* = 2.05, *p* = 0.04).

### 3.3 Attraction of *D. suzukii* to the Yeast *H. uvarum* Yeast is Reduced by Z4‐11Al

**FIGURE 2:**
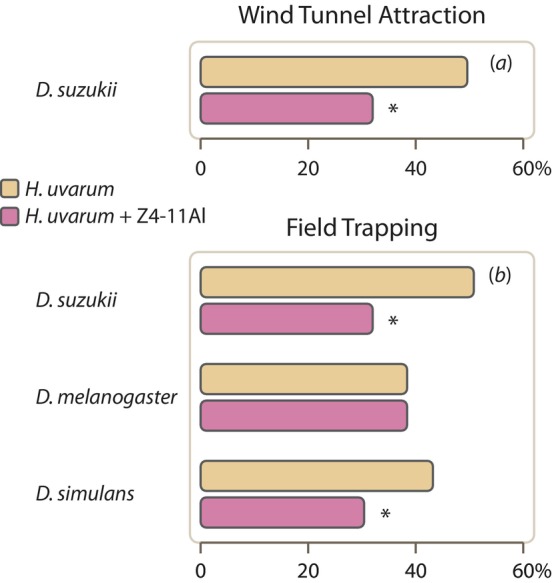
Effect of Z4‐11Al on attraction of *Drosophila suzukii* females to volatiles of the yeast mutualist *Hansenia uvarum*. (a) In a wind tunnel, *Z*4‐11Al was released at 10 ng/min, into an airstream passing through a wash bottle containing a fermenting *H. uvarum* culture. (b) In a field trapping assay, *Z*4‐11Al dispensers added to traps baited with *H. uvarum* reduced captures of *D. suzukii* and *D. simulans* , not captures of *D. melanogaster* . Asterisks indicate a significant difference (*p* < 0.05) following GLMM fitted with binomial and Poisson distributions for landing and trap captures, respectively.

*D. suzukii* is strongly attracted to the odour of fermenting *H. uvarum* yeast (Kleman et al. 2022; Spitaler et al. 2022; Rehermann et al. 2022). Releasing Z4‐11Al at a rate of 10 ng/min, into a plume of *H. uvarum* headspace in a wind tunnel, significantly reduced the attraction of *D. suzukii* (Figure 2a; *n* = 73, Z = −2.08, *p* = 0.03).

This was confirmed in a field trapping assay, where addition of Z4‐11Al to a *H. uvarum* bait significantly reduced trap captures of both *D. suzukii* and *D. simulans* (Figure 2b; *n* = 5, *p* = 0.02). Notably, Z4‐11Al did not have an effect on *D. melanogaster* attraction (Figure 2b).

## 4 Discussion

We confirm observations that egg‐laying in spotted wing Drosophila *D. suzukii* is reduced in the presence of a cognate species, *D. melanogaster* (Shaw et al. 2018; Kidera and Takahashi 2020; Kienzle and Rohlfs 2021; Tungadi et al. 2022, 2023; Rombaut et al. 2023) and show that this oviposition avoidance is mediated by the *D. melanogaster* female sex pheromone Z4‐11Al. When sharing food resources, larvae of *D. suzukii* are outcompeted by *D. melanogaster* (Dancau et al. 2017; Shaw et al. 2018; Rombaut et al. 2023), which provides an adaptive explanation for the response to heterospecific sex pheromone.

### 4.1 Pheromones Mediate Specific Mate Recognition and Reproductive Isolation

Pheromones serve specific mate finding and recognition, first of all. Sexual selection for efficient mate communication is, however, not an automatism for pheromone‐mediated interactions between different species. The recognition concept of species has called attention to the singular importance of mate communication adapted to the respective habitats of species, while it raised concern over the idea that the avoidance of hybrid matings is under strong selection, too, for its conceptual anthropomorphic bias and a lack of supporting data (Paterson 1985).

Meanwhile, experimental proof has been afforded for the interaction between sexual selection and species recognition through reinforcing natural selection against hybrid matings. Pheromone composition in the sibling species *D*. *birchii* and *D. serrata* differs between allopatric and sympatric populations, because contact between the two species in zones of geographic overlap gives rise to reproductive character displacement. And, the differences in pheromone communication observed over the natural range can also be generated by experimental sympatry in the laboratory (Higgie, Chenoweth, and Blows 2000; Higgie and Blows 2007, 2008).

Another strong argument for dual roles of pheromones is the widespread occurrence of interspecific pheromone antagonists in beetles and moths, where the same compounds reciprocally serve intra‐ and interspecific communication (Birch and Wood 1975; Lambert and Spencer 1995; Witzgall et al. 1996; Seybold et al. 2018). Pheromone analysis in moths frequently finds compounds that are attractive and antagonistic, respectively, within and between species, and such studies have even revealed cryptic species (Priesner and Baltensweiler 1987; Pelozuelo et al. 2004; Domingue et al. 2007; Bengtsson et al. 2014). Conversely, a lack of distinct pheromone barriers in geographically overlapping populations of sibling species is indicative of yet incomplete reproductive isolation (Saveer et al. 2014; Unbehend et al. 2014).

### 4.2 Pheromones Also Mediate Resource Partitioning

Chemically mediated interference between species is not restricted to premating sexual communication, but also concerns oviposition and consequently the use of larval food resources.

In tephritid fruit flies, host‐marking pheromones are known to deter oviposition in specific and heterospecific females, but these compounds do not serve mate communication (Prokopy, Reissig, and Moericke 1976; Aluja and Boller 1992; Sarles et al. 2015; Scolari et al. 2021). In contrast, *D. melanogaster* prefers to oviposit socially. A combination of microorganisms and pheromones deposited during oviposition, enhances the attraction of conspecific females (Wertheim et al. 2002, 2006; Golden and Dukas 2014; Venu et al. 2014; Durisko, Anderson, and Dukas 2014; Lin et al. 2015; Dumenil et al. 2016; Verschut et al. 2023).

We here show, for the first time, that the female sex pheromone of *D. melanogaster* Z4‐11Al deters egg‐laying in another species, *D*. *suzukii*. This demonstrates that the response to heterospecific sex pheromones is indeed under strong natural selection and underscores that resource partitioning via oviposition avoidance can further reinforce reproductive isolation.

The female *D. melanogaster* pheromone may even deter oviposition in other species. Our field trials show an antagonistic effect of Z4‐11Al on attraction to the fruit‐associated yeast *H. uvarum* , not only in *D*. *suzukii* but also in the sibling species *D. simulans* .

### 4.3 Z4‐11Al Mediates Communication Between *D. melanogaster* and *D. suzukii*

In *D*. *birchii* and *D. serrata* , changes in mate preferences in response to sympatry correlate with male cuticular hydrocarbons (CHCs). The behaviourally active compounds, however, have not yet been identified, possibly also because of a lack of clearcut, qualitative sex‐specific differences: CHCs in males and females differ only with respect to blend proportions (Higgie, Chenoweth, and Blows 2000; Howard et al. 2003; Higgie and Blows 2007, 2008). Likewise, the behavioural evidence that *D*. *suzukii* avoids host fruit occupied by *D. melanogaster* (Kidera and Takahashi 2020; Tungadi et al. 2022, 2023) has not yet been associated with specific chemicals. We here show, through a comparison of berries exposed to males and females, followed by experiments with synthetic pheromone, that the female *D. melanogaster* pheromone Z4‐11Al reduces egg‐laying in *D*. *suzukii*.

Pheromone interference between *D. melanogaster* and *D*. *suzukii* depends on fly density. Even males had an effect, when berries were pre‐exposed to a larger number of flies. A likely explanation is that mated flies carry both male and female pheromone, which are exchanged during mating (Bartelt, Schaner, and Jackson 1985; Everaerts et al. 2010; Frey et al. 2022), and deposited on substrates visited (Farine, Ferveur, and Everaerts 2012; Dumenil et al. 2016; Verschut et al. 2023).

Eggs laid by *D. melanogaster* females will further enhance oviposition avoidance by *D. suzukii*. Females coat their eggs with (*Z,Z*)‐7,11‐heptacosadiene (7,11‐HD) to prevent egg cannibalism (Narasimha et al. 2019). 7,11‐HD is the precursor of Z4‐11Al (Lebreton et al. 2017), and oxidation of 7,11‐HD deposited with eggs will continuously afford Z4‐11Al, which makes it difficult to test 7,11‐HD on its own.

Z4‐11Al also reduces *D. suzukii* flight attraction to *H. uvarum* , a highly attractive yeast mutualist associated with food sources (Hamby et al. 2014; Chakraborty et al. 2022; Kleman et al. 2022). This underscores that the interaction of fly pheromones and yeast volatiles, signalling suitable larval food resources, is understudied. A combination of specific social and habitat cues will further reinforce isolation between species.

In *D. melanogaster* , one variant of the olfactory receptor Or69a is tuned to Z4‐11Al, and the other to food odours (Lebreton et al. 2017). In *D. suzukii*, the orthologous DsuzOr69aB (Hickner et al. 2016; Walker et al. 2023) responds to Z4‐11Al and other unsaturated aldehydes (Cattaneo et al. 2023). Further functional characterization of the Or69a channel would be instructive, especially with respect to the question whether *D. suzukii* uses a pheromone of its own.

### 4.4 Competition Between Invasive and Established Species

The successful and rapid, worldwide range expansion of *D. suzukii* (Walsh et al. 2011; Cini et al. 2014; Asplen et al. 2015; Kwadha et al. 2021) is remarkable in view of its low competitiveness vis‐à‐vis the established cosmopolitan species *D. melanogaster* (Dancau et al. 2017; Shaw et al. 2018; Rombaut et al. 2023), which followed the human expansion out of Africa long ago (Lachaise and Silvain 2004; Mansourian et al. 2018; Arguello, Laurent, and Clark 2019).

Competitive interaction of larvae in fruit is expected to occur at the expense of *D. suzukii* larvae. Although *D. suzukii* infests earlier phenological stages than *D. melanogaster* , *D. melanogaster* may become attracted for oviposition before *D. suzukii* larval development is complete (Walsh et al. 2011; Keesey, Knaden, and Hansson 2015; Rodrigues et al. 2015; Rombaut et al. 2017; Silva‐Soares et al. 2017).

Flies in the wild encounter a wide range of plant hosts, and *D. suzukii* is likely to compete also with other drosophilid flies, in addition to *D. melanogaster* . Volatile signatures of flies may provide directions for spreading into new habitats and for colonizing competition‐free fruit space. Heterospecific competition is a niche dimension that relies on sensory perception, like mutualistic interactions during niche construction (Chakraborty et al. 2022).

### 4.5 Outlook on Integrated Management of *D. suzukii*

Field experiments with pheromones and fruit‐associated yeasts or yeast volatiles will be needed to further evaluate the role of Z4‐11Al in regulating niche partitioning and also its potential application in *D*. *suzukii* population management.

One promising approach for *D*. *suzukii* control is to combine larval or adult food attractants with killing agents (Mori et al. 2017). More recently, push‐pull strategies combining attractants and deterrents have received attention, where the deterrent components are general insect repellents, plant essential oils or volatiles from fungal fruit pathogens that deteriorate larval development (Cloonan et al. 2018; Wallingford, Cha, and Loeb 2018; Alkema, Dicke, and Wertheim 2019; Tungadi et al. 2023; Conroy et al. 2024).

Several deterrents could possibly be combined for enhanced efficacy. A drosophilid oviposition‐deterrent pheromone would confer the advantage of higher specificity, compared with plant or fungal metabolites, where its efficacy versus other deterrents and repellents is yet to be evaluated. Volatile compounds, such as Z4‐11Al, are inconvenient to formulate for season‐long field use, while the non‐volatile hydrocarbon precursor 7,11‐HD, that spontaneously oxidizes to afford Z4‐11Al, may be more suitable. Heterologous expression of biosynthetic enzymes for biotechnological production in yeast may even facilitate large‐scale production (Chertemps et al. 2006; Dam et al. 2024).

Taken together, an oviposition deterrent that is active only in drosophilid flies has the potential of becoming a crucial component in the integrated toolbox for *D. suzukii* management.

## Conflicts of Interest

The authors declare no conflicts of interest.

## Data Availability

The data of this study are shown in the manuscript. Raw data are available from the Dryad Data repository: https://doi.org/10.5061/dryad.z8w9ghxpd.
